# Immune Responses in Bladder Cancer-Role of Immune Cell Populations, Prognostic Factors and Therapeutic Implications

**DOI:** 10.3389/fonc.2019.01270

**Published:** 2019-11-20

**Authors:** Magdalene Joseph, Deborah Enting

**Affiliations:** ^1^Hayday Laboratory, School of Immunology and Microbial Sciences, King's College London, London, United Kingdom; ^2^Department of Uro-Oncology, Guy's Hospital, Guy's St Thomas NHS Foundation Trust, London, United Kingdom

**Keywords:** immunosurveillance, bladder cancer, genomic subtypes, Bacillus Calmette-Guerin, tumor microenvironment

## Abstract

Immunosurveillance, which describes the immunologically mediated elimination of transformed cells, has been widely accepted in the context of bladder cancer for many decades with the successful use of Bacillus-Calmette Guerin for superficial bladder cancer since the 1970s. With the emergence of checkpoint inhibitor blockade in the treatment of urothelial cancers, there has been a resurgent interest in the immunology of bladder cancer. The theory of cancer immunoediting proposes that the immune system has both pro-tumorigenic and anti-tumor effects, the balance between the two determining the progression of an individual tumor. However, whilst there is evidence for the action of various immune cell populations in bladder cancer, a cohesive picture of the immune response to bladder cancer and its driving forces are still lacking. Additionally, little is still known about the normal immune landscape of the bladder. Future progress in bladder cancer therapeutic approaches will require a strong foundation in understanding the immunology of this disease. This review considers the evidence for the role of the main immune cell populations, both innate and adaptive, in the immune response to bladder cancer. Recent research and overarching themes in the immune response to bladder cancer are explored. The minimal evidence regarding the normal immune landscape of the human bladder is also summarized to contextualize downstream immune responses. Of specific interest are the innate and myeloid populations, some of which are resident in the human bladder and which have significant effects on downstream adaptive tumor immunity. We discuss factors which restrain the efficacy of populations known to have anti-tumor activity such as cytotoxic T cells, including the constraints on checkpoint blockade. Additionally, the effects on the immune response of tumor intrinsic factors such as the genomic subtype of bladder cancer and the effect of common therapies such as chemotherapy and intravesical Bacillus Calmette-Guerin are considered. A significant theme is the polarization of immune responses within the tumor by a heavily immunosuppressive tumor microenvironment which affects the phenotype of multiple innate and adaptive populations. Throughout, clinical implications are discussed with suggestions for future research directions and therapeutic targeting.

## Introduction

Tumor immunosurveillance describes the ability of the immune system to recognize and eliminate transformed cells early in the tumorigenic process. By this definition, clinically detected cancers usually represent a failure of host tumor immunosurveillance. Whilst controversial when first proposed by Paul Erlich in the early 1900s, there is now a vast body of experimental and observational evidence suggesting an active role for the immune system in eliminating transformed cells (1, 2). More recently, growing awareness of some of the pro-tumorigenic actions of specific immune cell populations has led to a more comprehensive theory of cancer “immunoediting” (2).

**Figure 1:**
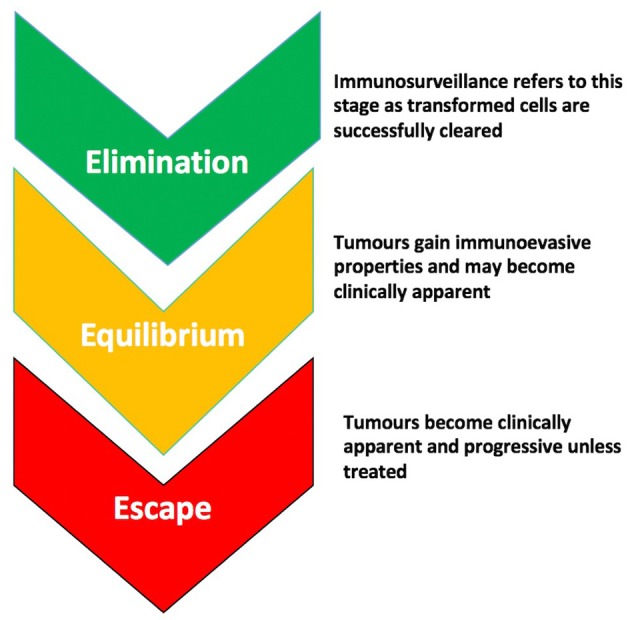
The stages of immunoediting. There are 3 stages proposed in the evolution of a tumor- “Elimination,” which if successful, leads to the abortion of the tumorigenic process through immunological mechanisms; “Equilibrium” during which the tumor gains immunoevasive properties allowing it to circumvent or suppress the anti-tumor effects of immune cells; and “Escape” during which the tumor overwhelms immune defenses and is usually progressive and fatal unless treated.

Immunoediting describes how pro- and anti-tumorigenic responses of the immune system, in concert with the properties of the tumor itself, can alter the clinical course of a tumor through “selecting” for less immunogenic clones. This theory proposes three stages in the evolution of a tumor- “elimination” (which if successful would abort the tumorigenic process); “equilibrium” where the tumor begins to gain immune-evasive properties that enable its survival; and “escape” whereby it overwhelms the immune system's defenses and is usually fatal unless treated (Figure 1). It is in the “equilibrium” and “escape” stages that tumors usually become clinically apparent, when some immunoevasive features have already developed. The older term “immunosurveillance” usually refers to the “elimination” part of this process. Little is known about proposed downstream immunoediting but evidence for immunosurveillance in bladder cancer is longstanding (3, 4).

In fact, a role for immunosurveillance in bladder cancer has been tacitly accepted since the 1970s when Morales et al. first demonstrated reduced tumor recurrence after intravesical Bacillus Calmette-Guerin (BCG) therapy (5). BCG treatment is now widely used in the treatment of superficial bladder cancer and arguably the most successful immunotherapy in use. However, the mechanisms underlying the anti-tumor response triggered by BCG and the general features of the immune response in bladder cancer are incompletely understood to this day. The advent of immune checkpoint-directed therapy makes it imperative to understand the mechanisms of immunosurveillance and immunoediting; and identify predictive immunological biomarkers for treatment and outcome.

Candidates for such biomarkers have included tumor infiltrating lymphocytes (TILs) and tumor somatic mutational burden (TMB). It has become clear over the past two decades that some cancers are more infiltrated by lymphocytes which appears to have prognostic significance in certain cancer settings (6, 7). It had been postulated that high lymphocyte infiltration may be secondary to a high TMB, and thus greater neo-antigen load. However, the evidence in bladder cancer suggests that it may be mutations in specific pathways rather than the quantity of somatic mutations which underlies immune infiltration (8). Additionally, there has been increasing interest in the innate and myeloid populations which are found abundantly in all tumors and are implicated in suppressing tumor immunosurveillance. Thus the search is on for more sophisticated biomarkers which integrate recent advances in tumor immunology.

In this review, we will explore what is known of the resident immune populations of the bladder. We will consider the role of both resident and recruited immune cell populations in the immune response to bladder cancer, focussing on their prognostic significance and the potential to therapeutically manipulate each population. We will also review the relevance to immune responses of recent advances in genomic and molecular subtyping and the effect of therapies as varied as chemotherapy, BCG and checkpoint blockade.

## The Immune Landscape of the Bladder in Health

There are few studies which have explored the bladder immune landscape in health and much of this knowledge comes from six mainly immunohistochemical analyses of bladder mucosa undertaken from the 1980s onward.

**Figure 2:**
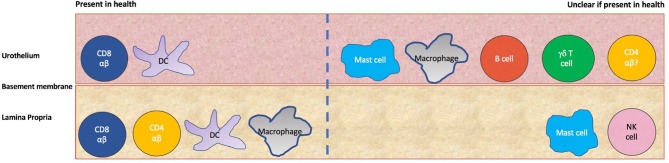
Immune populations in the human urothelium and lamina propria. On the left side are those populations whose presence is confirmed by more than one study, whilst on the right are those populations where uncertainty exists about tissue residence in health due to a lack of evidence or contradictory evidence from different studies.

These have used biopsies from small cohorts (5–13 subjects) of healthy control subjects or patients with non-bladder related pathologies- most recently, subjects were brain-dead, ventilated organ donors. There is no quantification of the different immune subsets in naïve human bladder, however, a picture can be built from the different qualitative studies (Figure 2 and Table 1). The earliest analyses suggest the urothelium is largely populated by HLA-DR +ve cells, some of which appear to be morphologically dendritic cells and some of which stain for the Langerhans' marker Cd1a, suggestive of Langerhans' like dendritic cells (15, 16). There is a smaller population of resident urothelial CD8 +ve T cells and immune cells in the urothelium were found to largely reside next to the basement membrane (10, 15, 16). In the lamina propria were more HLA-DR +ve cells, some with a macrophage-like appearance (15, 16). CD8 T cells and occasional CD4 T cells were also present in this layer (10, 15). Mast cells were found to reside in both layers using a toluidine dye staining method (11).

**Table 1:** Evidence for immune populations resident in naïve human and mouse bladder.

**Population of interest**	**Human urothelium (four studies)**	**Human lamina propria (single study)**	**Mouse whole bladder (single study)**
Neutrophils	Not described	Not described	Absent
B cells	Seen in 25% of cohort in one study, absent in smaller study previously	B cells not seen	B cells not examined
γδ T cells	Absent in earlier smaller study but seen in 55% of cohort in newer study	γδ T cells absent	γδ T cells present
NK cells	NK cells absent	Seen very occasionally (but used CD57 which is less specific marker)	NK cells present
CD4 T cells	Seen in 70% cohort in newer larger cohort but absent in 2 earlier studies	CD4 T cells present	CD4 T cells present
CD8 T	CD8 T cells present in all studies	CD8 T cells present	CD8 T cells absent
Dendritic cells	Seen in 3 studies, 2 of which found Langerhans type dendritic cells	Present with some Langerhans type dendritic cells	Dendritic cells present
Macrophages	Seen in newer cohort but absent in 2 older studies	Macrophages present	Macrophages and monocytes present
Mast cells	Mast cells seen in only study which investigated this	Mast cells seen	Mast cells and eosinophils seen

*A summary of the evidence for residence of different immune cell populations in the bladder. Evidence is presented separately for human urothelium, human lamina propria and whole mouse bladder. Evidence is derived from 6 studies published between 1986 and 2001 which are as follows: Mora-Bau et al. (9); Christmas et al. (10, 11); Gardiner et al. (12); Saint et al. (13); Cresswell et al. (14)*.

Whilst the earlier studies found an absence of γδ T cells, macrophages and CD4 T cells within the urothelium itself (10, 15, 16), the most recent analysis in 2001 found all three present in the urothelium in most donors with another small study finding CD3+ve CD8–ve T lymphocytes in the urothelium which could correspond to CD4 and γδ T cells (13, 14). Additionally, CD57+ve cells along the basement membrane which were characterized as natural killer (NK) cells in an earlier study were absent in the most recent study which used the same marker, now known not to be specific to NK cells (10, 15, 16). Whether these differences between earlier and later series are due to technological advances in immunohistochemistry, increased sample size or the nature of the donor cohorts is unclear.

The immune landscape in naïve mouse bladder is characterized more quantitatively by a single recent study (9). Though the method of whole bladder digest and flow cytometry does not allow for delineating the urothelium and lamina propria, it is a much higher sensitivity technique than those used for normal human bladder immunophenotyping. This showed that around 70% of CD45+ve cells in murine bladder are antigen presenting cells (APC). Macrophages constituted the largest APC population forming around 40% of CD45+ve cells, with dendritic cells a further 20% of CD45+ve cells. The rest was composed of a mix of CD4 T cells, NK 1.1+ve NK cells, γδ T cells and cKit+ve mast cells (9).

Interestingly, the CD8 T cells which are so prominent in naïve human bladder across multiple studies were completely absent in naïve mouse bladder suggesting that adaptive functions may be performed by γδ T cells instead which constituted around 2% of CD45+ve cells in the bladder (9). This potentially fundamental difference between murine and human bladder in health highlights the limitations of translating murine model findings to human disease.

However, this emerging picture of which populations normally reside in the bladder is essential for understanding downstream responses to bladder carcinogenesis. We will now consider the role of specific immune populations and their functional behavior in bladder cancer, with a focus on implications for prognosis and therapy.

## The Role of Immune Cell Populations in Bladder Cancer Immunosurveillance

Some key immune cell populations such as dendritic cells are already resident in the human bladder (7, 14, 15) and additional numbers of these can be recruited from the circulation. However, others such as neutrophils, FoxP3+ve regulatory T cells (T regs) and myeloid derived suppressor cells (MDSCs) are entirely recruited from the circulation in response to factors secreted by the tumor itself or surrounding immune cells. We will begin by considering cells of the innate immune system. These cells, which encompass some lymphoid populations, are still little understood or manipulated therapeutically despite being crucial gatekeepers and modulators of the immune response to cancer. Alongside sections on individual populations are sections discussing some of the most significant therapies and areas of research in bladder cancer and their relevance to the immune response in bladder cancer. These discussions include the relevance of genomic subtyping of bladder cancer and the impact of chemotherapy, BCG and checkpoint inhibition on immune responses.

## Macrophages and the Polarizing Effects of the Tumor Microenvironment

Macrophages have been detected consistently in immunohistochemical studies of healthy human bladder (13, 15, 16) and appear to have an overall pro-tumorigenic role. Multiple studies have correlated high tumor associated macrophage (TAM) counts with poor survival and poor response to treatment including chemotherapy and BCG therapy (17–19).

**Figure 3:**
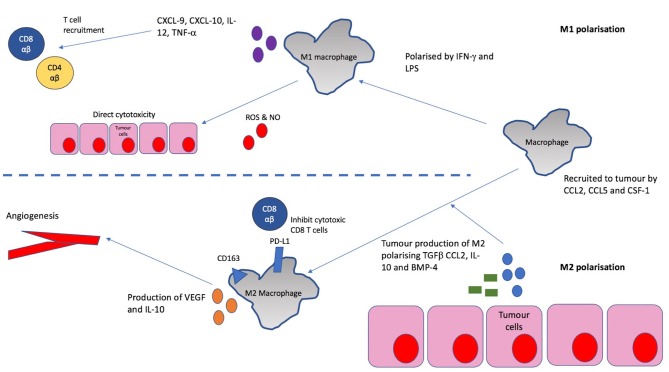
Recruitment and M2 polarization of macrophages in bladder cancer. Macrophages are recruited to the tumor by chemokines such as CCL2, CCL5, and CSF-1 which are often released by the tumor itself. In the tumor, they are polarized toward an M2 phenotype by the action of mediators including TGF-β, CCL2, IL-10, and BMP-4. This phenotype is characterized by CD163 expression, suppression of T cell responses, tissue remodeling and promotion of angiogenesis. This is mediated amongst other things by the production of vascular endothelial growth factor (VEGF) and IL-10, and the high expression of inhibitory checkpoint molecules such as PD-L1.

*In vitro*, macrophages are known to exhibit certain anti-tumor functions including phagocytosis, release of reactive oxygen species and secretion of inflammatory cytokines (20, 21). However, it is clear that these anti-tumor functions are largely lost in most cancers and TAMs have been found to be polarized to an immunosuppressive “M2” phenotype in multiple cancers (Figure 3) (22, 23). This is associated with CD163 expression, IL-10 production, angiogenesis and tissue remodeling in murine models of various cancers- all of which can contribute to increased tumor growth and metastasis (24). In fact, one study in human bladder cancer found that an increasing CD163/CD68 ratio (and therefore, increased M2 polarization as CD68 is a pan-macrophage marker) correlates with higher disease stage and vascularity (25) suggesting M2 polarized macrophages are responsible for the angiogenesis seen in bladder tumors.

Whilst, IFN-γ and LPS are capable of polarizing macrophages to a “M1” pro-inflammatory phenotype associated with cytotoxicity (24), these are rarely the predominant players in the tumor microenvironment. Production of M2 polarizing cytokines such as CCL2, IL-10, and TGF-β by bladder tumors has been demonstrated in various *in vitro* studies (26–28) and IL-10 production by bladder tumor cells has been shown to induce an immunosuppressive monocyte phenotype (Figure 3) (29). There may also be a role for bone morphogenic proteins (BMPs) produced by bladder tumors in M2 polarization, with a recent study finding BMP-4 induces a M2 macrophage phenotype in bladder cancer *in vitro* (30).

In addition to their effects on tissue remodeling and tumor angiogenesis, M2 macrophages promote tumorigenesis partly through their effects on the adaptive immune system in their function as antigen presenting cells (APCs). It has been demonstrated in co-culture experiments that IL-10 production by bladder cancer cells leads to increased PD-L1 expression on monocytes and downstream suppression of T cell immune responses (29). Additionally, M2 macrophages lack production of chemokines such as CXCL9 and CXCL10 which recruit Th1 lymphocytes with anti-tumor activity (23). This may explain findings in a cohort of 296 patients where the strongest association with poor survival was predicted by a high CD68/CD3 ratio (31) suggesting that macrophage high tumors may correlate with poor T cell infiltration.

In fact, a recent study categorized tumors on the basis of two stromal immune infiltration patterns and found that the subtype with low macrophage infiltration and high cytotoxic lymphocyte infiltration was associated with improved survival with the presence of these populations inversely correlated (17). Thus, whilst macrophages do not directly influence clonal selection in tumors and immunoediting, they appear to broadly suppress adaptive immunosurveillance and create a tumor favoring microenvironment in bladder cancer. Any therapeutic strategy which aims to improve on current response rates, has to address this key axis of immunosuppression.

## Genomic Subtypes of Bladder Cancer and Immunosurveillance Implications

Also greatly affecting immune cell infiltration into tumors is the intrinsic genomic subtype of bladder cancer which affects prognosis as well as response to therapies (32). The genomic subtype is often a reflection of the layer or tissue of origin of the tumor. Multiple sub-classifications have been proposed over the years based on different cohorts of patients and a recent attempt to reach a consensus has identified 6 main subtypes in muscle invasive bladder cancer, some of which are more immune cell infiltrated than others (33). Basal/squamous tumors, the commonest subtype (~35%), arise from the basal layer of the urothelium and are enriched for mutations in tumor suppressors such as p53 and RB1 (33).

Despite being heavily infiltrated with immune cells, including cytotoxic T cells and NK cells expressing high levels of inhibitory checkpoint receptors, these tumors do not respond to immunotherapy as well as less heavily infiltrated tumors (33). This suggests that the local tumor environment might be too immunosuppressive to overcome with single agent immunotherapy alone. A recent study analysing immune subset infiltration in bladder cancer using bulk transcriptomes (CIBERSORT) found that M2 macrophage infiltration was associated with the basal subtype of bladder cancers and a higher histological and pathological grade suggesting that M2 macrophages may be responsible for the poor response to immunotherapy seen in this group and thus a target for future intervention (34).

On the other end of the spectrum, the luminal unstable subtype, which arises from the luminal layers of the urothelium and is the subtype with the highest mutational load, does not demonstrate any associations with an immune infiltrating signature (33), despite the possibility of more neoantigens within the tumor. However, this subtype shows greater benefit from checkpoint inhibitor immunotherapy than the heavily immune infiltrated basal squamous subtype (33). These findings suggest that susceptibility to immune therapies does not correlate directly with mutational load or depend simply on the baseline level of immune infiltration (33). It is clear one has to consider the interplay between the tumor and multiple immune populations which are capable of polarization toward pro- or anti-tumorigenic actions.

Neutrophils are one such population which demonstrate similar pro-tumorigenic polarization to macrophages in most disease settings. They are of particular interest in the context of BCG therapy where this usual pro-tumorigenic polarization appears to be reversed by an external intervention highlighting the importance of immune modulating therapies.

## Neutrophils, the Neutrophil-lymphocyte Ratio and BCG Immunotherapy

Neutrophils are absent in healthy bladder, but are found in higher proportions in the circulation in cancer patients and abundantly in bladder tumor where they appear to have a largely immunosuppressive effect unless their activity is modulated by concomitant therapies (35). Numerous studies have examined circulating neutrophil-lymphocyte ratio (NLR) as a possible biomarker to predict prognosis or response to treatment in bladder cancer. A systematic review in 2016 analyzed NLR in urothelial cancer, covering 23 studies and 6,240 patients (36). It found that a high NLR appeared to correlate with worse overall, recurrence-free and cancer specific survival (36) with similar findings in a meta-analysis of NLR as a prognostic marker in non-muscle invasive bladder cancer (37). It has also been shown that higher tumor infiltrating neutrophil count and NLR both predict advanced pathological stage and poorer survival confirming the link between what is seen in the circulation and the tumor milieu (38).

One of the mechanisms by which this circulating neutrophilia and accumulation in tumor develops is likely related to the direct release from tumor cells of cytokines such as CXCL1, CXCL5 and, in particular, IL-8 which is known to be a potent chemoattractant for neutrophils. IL-8 is known to be constitutively produced by urothelium in health (39) but many human bladder cancer cell lines overexpress the cytokine (40, 41). In humans, urinary levels of IL-8 have been shown to correlate with the presence of transitional cell carcinoma and its stage (42) and circulating levels of IL-6 and IL-8 have been correlated to NLR in a cohort of 121 patients (43).

Evidence from non-bladder cancer mouse models suggests that the local cytokine milieu in the tumor has a significant effect on neutrophils, with TGF-β polarizing toward an immunosuppressive phenotype (35, 44). As bladder cancer is known to produce higher levels of TGF-β than normal tissue (28), this might be a mechanism by which tumor infiltrating neutrophils are polarized toward immunosuppression and explain the correlation between tumor infiltrating neutrophils and poor survival. These immunosuppressive “N2” polarized neutrophils, which express high levels of arginase (45), are known to assist invasion, metastasis and angiogenesis in other cancers through the release of proteases such as neutrophil elastase and matrix metalloproteinase-9 (MMP-9) (35). *In vitro* experiments using “N2” neutrophils polarized by co-culturing with bladder cancer cell lines demonstrated that these neutrophils enhanced the invasiveness of the bladder cancer cells in “*in vitro*” assays (46).

However, tumor infiltrating neutrophils can take on a completely different role following therapeutic manipulation or a change in the tumor environment. Blocking the effects of TGF-β with an orally administered small molecule inhibitor in a mouse tumor model led to the accumulation of neutrophils with a cytotoxic and anti-tumor phenotype compared to those in control mice (44). In fact, the actions of neutrophils can be essential in some settings.

BCG therapy for superficial bladder cancer is one of the earliest prototypic immunotherapies where neutrophils are essential to the anti-tumor effect (47, 48). Often used to treat superficial non-muscle invasive bladder cancers (NMIBC), BCG is used at a stage where the tumor burden, and thus TGF-β levels and any adversely polarizing effects on neutrophils, is minimal. The uptake of mycobacterium by the urothelium and resident antigen presenting cells triggers release of cytokines such as IL-6, IL-8, GM-CSF, and TNFs which induce a rapid and heavy infiltration of neutrophils into the bladder within hours of instillation in patients undergoing BCG therapy (48, 49).

**Figure 4:**
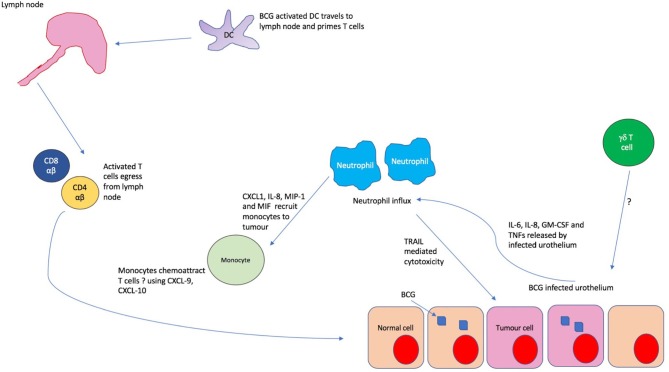
The immunological cascade in BCG treatment. Uptake of BCG by bladder urothelium triggers release of IL-6, IL-8, GM-CSF and tumor necrosis factors (TNFs) which recruit copious neutrophils to the bladder. These neutrophils chemoattract monocytes to the tumor environment through the release of CXCL1, IL-8, MIP-1, and MIF. It is thought that these monocytes subsequently induce T cell migration, possibly through the release of chemokines such as CXCL9 and 10. In the meantime, BCG activated dendritic cells migrate to the lymph nodes and prime antigen naïve T cells which then egress from the lymph node and are recruited to the tumor. Whilst γδ T cells are found in large numbers in the urine post BCG, it is unclear how they are recruited to the bladder.

In fact, mice depleted of neutrophils show no therapeutic benefit from BCG when compared to untreated controls, with an absence of CD4 T cell influx (47) which is known to be essential to the anti-tumor response. The mechanism of action in murine models appears to depend on indirect recruitment of T cells through the recruitment and activation of monocytes, mediated through the release of cytokines such as CXCL1, IL-8, MIP-1, and MIF (47). Thus, it appears that neutrophils mediate the influx of later immune mediators of the anti-tumor BCG response such as monocytes and CD4 T cells (Figure 4). In addition, there is emerging evidence of direct anti-tumor effects through the release of apoptotic mediators such as tumor necrosis factor-related apoptosis- inducing ligand (TRAIL) which is known to be selectively cytotoxic to tumor cells (50). It has been shown that urinary excretion of TRAIL positively correlates with response to BCG in humans, with TRAIL demonstrating direct cytotoxicity of bladder tumor cells *in vitro* (51).

Additionally, there is *in vitro* evidence that BCG activated macrophages exhibit pro-inflammatory and tumoricidal properties, demonstrating the favorable effects of this therapy on multiple innate populations (21). It is clear from the evidence considered that triggering innate immune responses can have clinically significant effects on downstream adaptive immunity. Such repolarization of usually immunosuppressive populations toward anti-tumor responses raises the tantalizing prospect of future therapies targeting innate populations.

## Myeloid Derived Suppressor Cells and the Immunological Effects of Chemotherapy

Closely related to monocytes and neutrophil precursors, myeloid derived suppressor cells are immature myeloid cells which show immunosuppressive properties and are absent in healthy individuals. It is thought that the chronic inflammatory milieu of cancer induces the release of immature myeloid cells from the bone marrow and they are often found amongst the mononuclear cell fraction in peripheral blood and in tumors of various types (52). The granulocytic subtype, G-MDSC, identified as CD11b+veCD14–veCD15+ve are the predominant subtype found in malignant settings with a minor monocytic subtype, M-MDSC, which is CD11b+veCD14+veHLA–DR–ve/lowCD15–ve (52). CD33, a marker of immature myeloid cells can additionally be used to characterize these cells with G-MDSC expressing low levels and M-MDSC expressing high levels. The main barrier to characterizing the role of these cells has been the lack of consistency across studies in the way these cells are identified using surface markers (53). However, there is ample evidence that immature myeloid cells have adverse effects in bladder cancer.

High numbers of peripheral blood MDSC were found to adversely correlate with stage, grade and prognosis in bladder cancer by Yang et al. in 2016, though the specific subtypes of MDSC were not analyzed (54). Previously, Eruslanov et al. had shown that G-MDSCs were expanded in the PBMC fraction of bladder cancer patients compared to healthy individuals, whilst there was no similar expansion of the M-MDSC subtype (55). These G-MDSCs were capable of significant pro-inflammatory cytokine production and inhibited *in vitro* T cell proliferation by inducing FoxP3+ve regulatory T cells (55).

These patterns are further reflected in the bladder tissue itself where G-MDSC were the predominant MDSC subtype present in an analysis of fresh bladder tissue (56). The degree of infiltration of G-MDSC negatively correlated with the CD8 T cell infiltration in another study (57), in keeping with the T cell suppressive effects observed previously by Eruslanov et al. The MDSC present in bladder tumor have been shown to express high levels of immunosuppressive molecules such as Arginase 1, inducible nitric oxide synthases (iNOS) and PD-L1 and directly suppress T-cell proliferation reflecting their phenotype in the peripheral blood (58).

Whilst, we have mostly considered G-MDSC, a role for M-MDSC in the context of response to BCG has been suggested by Chevalier et al. who found a T-cell to MDSC ratio of <1 correlating with a poorer recurrence free survival post treatment (59). Recruitment of these cells may represent one of the factors underlying BCG failure in some patients.

Interestingly, treating PBMCs derived from patients with bladder cancer with cisplatin has been shown to selectively deplete G-MDSC (57). Additionally, T cells cultured with cisplatin-treated peripheral blood G-MDSCs exhibited less inhibition of their tumor apoptosis promoting capabilities than those cultured with untreated G-MDSCs (57). This suggests one of the anti-tumor effects of cisplatin may be mediated by reshaping the immune compartments in bladder cancer and suppressing G-MDSC proliferation and function. Immune enhancing properties of chemotherapy have been observed in other cancers and this is an exciting area for further research (60).

## Natural Killer Cell Subsets and γδ T Cells

Thus far we have considered the innate, myeloid immune populations and their usually pro-tumorigenic role in bladder cancer, in the absence of additional therapies. Bridging the divide between the innate and adaptive immune system are lymphoid cells of the innate immune system and unconventional T cells such as natural killer cells (NK) and γδ T cells. These are thought to be one of the earliest effectors of the anti-tumor response and do not depend on MHC-restricted antigen presentation for activation (61). These cells are capable of recognizing stress ligands which are overexpressed by tumor cells, including MICA, MICB and the ULBP family, and respond with perforin-mediated cytotoxicity or IFN-γ production (2, 61). IFN-γ is capable of polarizing other immune populations, including macrophages, toward a type 1 anti-tumor response.

Evidence for the role of natural killer (NK) cells in bladder cancer is patchy and inconclusive at best and they do not appear to be a resident population in healthy human bladder (10, 62). However, a retrospective study of patients with non-muscle invasive bladder cancer (NMIBC) found that baseline NK cell infiltration was significantly higher in the group of patients who had recurred at 2 years follow up compared with disease free patients (63). There was also an association between tumor size and increased NK cell infiltration (63) which appears to suggest lack of efficacy, or worse, an adverse role.

Surprisingly, a more recent flow cytometric analysis of fresh tissue from patients with both NMIBC and muscle invasive bladder cancer (MIBC) has suggested that improved survival may be linked to the presence of CD56^bright^ NK cells within the tumor which are found in much smaller numbers than their CD56^dim^ counterparts. These cells are functionally active compared to the reduced cytokine secreting capacities of the CD56^dim^ subset (64). This might explain the findings of the previous study which did not distinguish between the subsets in their analysis.

NK cells are also of particular interest in the context of BCG where a lack of efficacy of BCG in NK-cell deficient beige mice has been reported (65). However, it appears the presence of monocytes is essential, at least for the CD56^dim^ population. Bisiaux et al. investigated the dependence on monocytes for activation by BCG and found that γδ T cells and CD56^bright^ NK cells appeared to be capable of activation by BCG alone, in the absence of monocytes, whereas αβ T cells and CD56^dim^ NK cell activation was dependent on monocytes (66). Thus it appears that there are two NK cell populations with very different behaviors and associations in bladder cancer and these will have to be studied individually in future analyses.

NK cell mediated cytotoxicity is dependent on receptors such as NKG2D which bind stress ligands that are overexpressed on tumor cells. Experiments blocking NKG2D show reduced cytotoxicity of NK cells against bladder cancer lines demonstrating the importance of this mechanism of tumor recognition (67) and these receptors are highly expressed on other cytotoxic populations including CD8 αβ T cells and NKT cells.

One lymphocyte population bearing high levels of NKG2D and of particular interest in the context of BCG are γδ T cells which are a resident population in naïve mouse bladder and may be resident in healthy human bladder (9, 62). These cells bear a rearranged γδ TCR but are capable of non-MHC restricted activation, cytotoxicity and cytokine production. Multiple studies have shown that this otherwise small lymphocyte population is enriched in the urine and tissue of patients undergoing BCG therapy for bladder cancer and the level of increase seems to correlate with a positive outcome from therapy (68, 69). This has been further bolstered by mouse studies which have shown a lack of effect of BCG in γδ deficient mice, which appears to be dependent on their production of IL-17 and recruitment of neutrophils (70). Given that γδ T cells can be activated by BCG in a monocyte-independent fashion as discussed earlier, this positions them center stage in the response to BCG treatment.

Whilst γδ T cells can also be activated by NKG2D ligands, another possible mode of activation in this context is through the Vγ9δ2 TCR which is activated in a phosphoantigen dependent manner, with phosphoantigens being abundant in BCG. In support of this hypothesis, γδ T cells produced in urine post-BCG therapy were enriched for this subset in one study (69). Promisingly, a survival benefit from intravesical administration of this subset of human γδ T cells has been demonstrated in a murine orthoptic bladder cancer model (71).

The role of CD56^bright^ NK cells and γδ T cells outwith of BCG therapy remains unclear but the evidence for their independent activation by BCG raises the possibility of enhancing BCG therapy by using the potentiating effects of these cells. γδ T cells are currently being trialed as an adoptive therapy in various cancers and may yet enter the realms of bladder cancer therapy.

We will now consider the responses of the adaptive immune system, beginning with the classical gatekeepers to an adaptive immune response, dendritic cells.

## Dendritic Cells

Dendritic cells (DC) are potent mediators of adaptive immunity through their ability to present antigen to and activate T cells. They constitute around 20% of CD45+ve cells in the naïve mouse bladder and dendritic appearing HLA-DR +ve cells have been identified in healthy human bladder suggesting residence (9, 15, 16). These cells constantly sample their environment for antigens and, in the context of danger signals such as heat shock proteins released from necrotic tumor cells, they can be activated and migrate to the lymph nodes where they prime naïve T cells (20).

Whilst a relatively understudied area in bladder cancer, there is evidence that dendritic cell number or function may be affected in the context of bladder cancer. Few studies, mainly examining myeloid subset DCs, have shown that peripheral blood dendritic cell counts appear to be reduced in patients with bladder cancer compared to healthy individuals (72, 73). One study found that surgery increases this peripheral blood DC count relative to baseline in those with superficial disease (74). This suggests that dendritic cells may be depleted from the blood by the tumorigenic process.

In support of some depletive mechanism would be the observation that high levels of tumor infiltrating DCs in human bladder cancer predict progression to muscle invasion suggesting that DCs may be a significant but unhelpful presence in bladder cancer (75). Dendritic cells have been shown to be a significant part of the tumor infiltrate, constituting around 17% of CD45+ve cells within the tumor in one study (76). These DCs appeared phenotypically immature with low CD80 and CD86 expression and this immaturity persisted even in higher stage tumors that had greater numbers of infiltrating DCs (76).

This blocking of maturation was further confirmed by Beatty et al. who found immature DCs in the tumor and urine of patients with superficial disease pre-BCG (77). All of this suggests a functional deficit in dendritic cells in the context of bladder cancer and experiments have shown that monocyte derived dendritic cells adopt an immature phenotype when co-cultured with bladder cancer cells (78, 79).

Malignancy associated glycan, Sialyn-Tn, has been implicated in this process and shown to induce an immature phenotype in human monocyte derived dendritic cells *in vitro* (79). These low HLA-DR, CD80, and CD86 DCs were impaired in their ability to activate T cells. The T cells were, in fact, skewed toward a FoxP3-high, IFN-γ- low regulatory phenotype. This skewing of the immune response and blocking of DC maturation was partially reversed by blocking of Sialyn-Tn antigens, CD44 and MUC1, suggesting a causal relationship between specific glycan expression by tumors and inhibition of DC maturation (79).

However, there are likely to be a multitude of inhibitory influences on DCs in the tumor microenvironment and the upregulation of the JAK2/STAT3 pathway in tumors has been implicated in blocking DC maturation *in vitro* (78). An inhibitor of JAK2, AG490, was found to partially reverse maturation block of DCs providing a possible therapeutic target (78).

Whilst recent approaches to overcoming such blocks have focused on genetically modified DCs or *ex-vivo* antigen loading and activation, these have had modest effects *in vitro* and have not translated into significant clinical outcomes. Lapuleucel-T, a dendritic cell based therapy using monocytes activated with recombinant GM-CSF linked to a HER2 peptide showed no statistically significant overall survival benefit or disease free survival benefit in a cohort of high risk HER2+ve bladder cancer patients in a phase 2 study (80, 81).

However, there might be a role for DCs in potentiating BCG therapy. In a small cohort of 12 patients undergoing BCG therapy for superficial disease, Beatty et al. found that the 6 patients who responded to BCG had a trend toward increased urinary DCs post treatment with the reverse in the non-responding group (77). A role for DCs in the response to BCG is further suggested by a study which found that myeloid DC numbers in the blood and urine rose in response to BCG treatment (72).

Given the previous evidence of maturation block of DCs in bladder cancer, this suggestion of a positive effect of DCs on clinical outcome might be explained by findings that BCG induced maturation of peripheral monocyte derived DCs which were then able to activate natural killer T cells (NKT) and γδ T cells to lyse bladder cancer cells (82). Thus, BCG may be capable of converting the immunosuppressed, immature DC phenotype to an anti-tumor one. In fact, when BCG was added to co-cultures of PBMCs and bladder cancer cells, minimal inhibition of tumor growth was observed unless BCG-infected DCs were added (83).

Thus, like macrophages and neutrophils, the anti-tumor capabilities of dendritic cells too appear largely suppressed by the tumor microenvironment with BCG possibly providing a way to reverse this inhibitory effect. It appears that the tumor is capable of steering these key myeloid populations toward actions which globally suppress immunosurveillance of the tumor. As we will see in the next section, such immunosuppressive polarization also affects the lymphocyte populations of the adaptive immune system.

## CD4 Helper T Cells and the Th1/Th2 Axis

CD4 T cells are resident in the murine bladder and may constitute a resident population in healthy human bladder (9, 10, 15, 62). However, they are most potently recruited to the immune response through encountering activated dendritic cells in the lymph nodes (84). On encountering their cognate TCR ligand on an activated DC, naïve CD4 T cells can acquire different phenotypes which depend on the cytokine milieu and phenotype of the dendritic cell. Murine and human *in vitro* experiments, as well as mouse models have established that a Th1 phenotype is acquired in the presence of IL-12 ± IFN-α production and is characterized by high IL-2 and IFN-γ production and tumor suppressing responses (84, 85). On migration to the tumor milieu, such Th1 polarized cells can promote macrophage and CD8 T cell mediated cytotoxicity through IFN-γ production.

The significance of Th1 cells to the anti-tumor response is highlighted by a recent study investigating the effect of checkpoint blockade in a murine bladder cancer model. Analysis of the immune infiltrates from tumor and lymph nodes post treatment revealed an expansion of IFN-γ producing CD4 T cells (Th1) and the neutralization of IFN-γ abolished the anti-tumor effect of checkpoint blockade suggesting the key role for these cells and this cytokine (86). Additionally, a Th1 biased response is known to be essential to successful BCG therapy (87, 88) with a recent study demonstrating a transient but significant recruitment of CD4 helper T cells to the bladder in a murine model- recruitment which far outstripped that of cytotoxic CD8 or regulatory FoxP3 T cells (89).

In contrast, Th2 polarization which is characterized by the promotion of humoral immunity requires IL-4, the source of which is less clear, and is characterized by IL-4, IL-10, and IL-13 production, the net effect of which is to suppress cytotoxic immune responses (84, 85). CD4 T cells are additionally capable of adopting a regulatory phenotype (Treg) characterized by FoxP3 and CD25 expression or an IL-17 producing Th17 phenotype (90, 91). However, as these are often induced peripherally in the context of cancer, we will consider these in a separate section.

No less important a role for CD4 T cells is their reciprocally activating effects on dendritic cells through the CD40L-CD40 axis, further potentiating the antigen presenting capabilities of DCs and promoting cytotoxic lymphocyte activation (84). Whilst CD4 T cells are detected in bladder tumors, little is known about their functional potential and many studies are limited by the lack of functional phenotyping of the different subclasses.

A higher CD4 T cell density within the tumor was found to correlate with a poor prognosis in a study of 131 patients with NMIBC and, suggesting a similar adverse correlation for CD4 T cells, a high CD3/CD4 ratio was found to correlate with better survival in MIBC in an analysis of 4 publicly available genomic datasets (92, 93). However, both of these studies are limited by the aforementioned lack of functional phenotyping of the CD4 T cells present.

A recent study of methylation patterns of CD4 T cells from the tumor in patients with bladder cancer showed that a higher stage was correlated with increased methylation (and thus reduced expression) at the IFN-γ locus (94). Conversely, patients with a complete response to neoadjuvant chemotherapy showed significant hypomethylation at loci related to all functional types, but most prominently at the IFN-γ locus confirming the anti-tumor role expected of Th1 polarized cells (94).

However, the picture in untreated or progressive bladder cancer is one of dominant Th2 polarization with Satyam et al. finding IFN-γ and IL-2 levels to be significantly lower in the blood of patients with bladder compared to healthy individuals with the inverse true of Th2 cytokines IL-4, IL5, and IL-10 (95). Intriguingly, a role in Th2 polarization has been suggested for the little known double positive CD4+veCD8+ve T cell population which was found to be expanded in the blood of patients with bladder cancer in a recent study. These cells were shown to be capable Th2 cytokine production and inducing Th2 polarization in naïve CD4 T cells (96).

Whilst suggestive of an adverse correlation with Th2 skewing, the evidence above is inconclusive for the role of Th1 and Th2 helper CD4 cells in bladder cancer. Similarly ill-defined is the role of the CD4+ve FoxP3+veCD25+ve regulatory T cell which we will consider next.

## FoxP3 Regulatory Cells and Th17 Helper T Cells

Regulatory FoxP3+veCD25+ve CD4 T cells (Tregs) are known to have significant tumor promoting effects in many cancers and can be of thymic origin or induced locally through the actions of TGFβ in concert with other immunosuppressive cytokines such as IL-10 (97, 98). In fact, blocking IL-10 and TGFβ has been shown to reduce the induction of CD25+ve regulatory T cells *in vitro* by a bladder cancer cell line (26). Such Tregs are capable of suppressing immune responses to cancer, often through their suppression of cytotoxic T cells and constitute a significant percentage of TILs in bladder cancer- a median of 17% across samples in one study (99). However, a clear role for regulatory T cells in bladder cancer is unclear with some studies suggesting a positive correlation with clinical outcomes.

Winerdal et al. used immunohistochemistry to analyse 37 cystectomy specimens ranging from pT1 to pT4 disease and found a higher infiltration of FoxP3 cells correlated with improved survival (100). They suggested this may be secondary to FoxP3 being upregulated on T cell activation, thus acting as a marker of activated T cells, rather than regulatory T cells alone. However, in support of a true anti-tumorigenic role, the same group found an inverse correlation between “true” Tregs at the invasive front of urothelial cancers and the expression of matrix metalloproteinase 2 (MMP2) which is produced by tumor cells and macrophages and promotes tumor invasion (101). Within their cohort, which included all T stages of disease, presence of Tregs at the invasive front also correlated positively with survival. The group found that these Tregs bore epigenetic marks of Treg differentiation and stably expressed FoxP3 suggesting they are “true” Tregs (101).

However, numerous studies have found negative correlations between FoxP3 T regulatory cells and survival including one study of 115 cases of NMIBC which found an inverse correlation between FoxP3 cell frequency within the tumor (as % of CD3+ve cells) and recurrence-free survival (99). Additionally, higher FoxP3 infiltration in the stroma around the tumor was shown to predict a shorter recurrence free survival post-BCG treatment for NMIBC (102). In the context of MIBC, a higher CD8/FoxP3 tumor infiltrating lymphocyte TIL ratio predicted better response to neoadjuvant chemotherapy though the density of either considered alone did not show this same correlation, with another study finding the same correlation with CD8/FoxP3 TIL ratio and survival post cystectomy (103, 104).

It is difficult to reconcile these very contradictory findings which show different prognostic associations for Tregs. This may be due to variation in the location of the Tregs being measured- whether within the tumor or surrounding the tumor. It is also possible that low level FoxP3 or CD25 expression by activated non-regulatory T cells may cloud the picture when additional makers are not used to fully phenotype regulatory T cells.

An additional question surrounds the antigen specificity of possible suppressive responses by Tregs, with a recent murine study suggesting that suppression of naïve CD4 responses by Tregs is antigen specific and related to removal of peptide-MHC II complexes from the surface of dendritic cells (105). Whilst no evidence exists for this mechanism of Treg mediated suppression at present in humans, it might explain how immunoediting operates through antigen specific immunosuppression to select for specific tumor clones.

One important cell type to consider in concert with regulatory T cells is the pro-inflammatory Th17 subgroup of CD4 T helper cells which are characterized by the expression of the RORγt transcription factor. Unlike, Tregs which can have a thymic origin, these cells are exclusively induced in the periphery and their differentiation appears to be reciprocally regulated with respect to Tregs. In humans, IL-1β and IL-6 are the key cytokines implicated in their induction (90). Th17 cells have been found to be enriched in bladder tumor relative to peripheral blood from patients and healthy individuals, suggesting a local induction of this sub-group by bladder tumor (90).

However, the role of Th17 cells in bladder cancer is still largely unstudied. In mice, responses to BCG have been shown to depend on IL-17 production, albeit by γδ T cells (70). However, in the absence of additional therapies, IL-17 may be pro-tumorigenic through its angiogenic effects amongst others and IL-17 knockout mice exhibit reduced growth of orthoptic bladder and melanoma tumors (106).

Thus, the role and prognostic significance of Tregs and Th17 cells in bladder cancer remains unclear. One population where there is no doubt about their prognostic significance is CD8+ve cytotoxic T cells. The evidence overwhelmingly suggests a favorable correlation with outcomes and their antigen specificity implies a degree of immunoediting and we will discuss these in this final section.

## Cytotoxic T Cells and the Promise of Checkpoint Inhibitor Immunotherapy

Cytotoxic CD8 T cells have been the focus of immuno-oncology for the past decade with the success of checkpoint inhibition in multiple cancers, including bladder cancer. Studies suggest they are a resident population in human bladder and they are present in high densities in some molecular subtypes of bladder cancer (9, 10, 33, 62). CD8 T cells in the tumor are believed to be specific for tumor associated antigens and a recent analysis of the TCRβ repertoire (both CD4 and CD8 T cells) in MIBC correlated low TCR diversity and high neoantigen load with improved survival suggesting that tumor antigen specific immune responses are a key part of anti-tumor immunity (107).

Whilst some studies have suggested a favorable prognostic significance for the presence of CD8 T cells in bladder cancer (108), others have suggested that this association appears to be modulated by the presence of other immune cell populations, including regulatory FoxP3+ve T cells. As previously mentioned, a favorable response to neoadjuvant chemotherapy was found to be predicted by a high CD8/FoxP3 ratio in the tumor, but not the level of infiltration of either population alone (103).

We have discussed earlier the suppression of T cells responses by expression of PD-L1 on macrophages and monocytes within the tumor. The significance of this pathway is strengthened by studies demonstrating that CD8 T cells infiltrating bladder tumors often show high expression of PD-1 (109, 110), the receptor for PD-L1, which is known to be characteristic of an exhausted phenotype in the context of chronic antigenic stimulation. This chronic antigen exposure may result from the inhibition of their cytotoxic potential by tumor cells with one study showing that urothelial cancer supernatants suppressed perforin expression in CD8 T cells. This was associated with upregulation of TGFβ signaling pathways, suggesting that this is TGF-β mediated (111). Additionally, the immunosuppressive environment of the tumor with a lack of IFN-γ and IL-12 production and production of immunosuppressive cytokines by multiple immune populations can directly suppress CD8 T cell responses (2).

The exhausted phenotype of CD8 T cells has been correlated with a shorter recurrence free survival in a recent study which examined PD-1 expression on urine derived lymphocytes pre-cystectomy (112). However, this exhaustion can be exploited therapeutically and the PD-1/PD-L1 axis in bladder cancer has been targeted with checkpoint inhibitor blockade with some success over the past 5 years. Overall, response rates range from 15 to 50% dependent on patient selection and biomarker enrichment (113). Despite these findings, the prognostic and predictive value of expression of PD-1 or PD-L1 within the tumor remain incompletely defined (114).

There is also the untested possibility that CD8 T cell mediated cytotoxicity is one mechanism by which immunoediting operates within bladder cancers. It is known that many bladder cancers downregulate the expression of MHC class I and II proteins (115, 116), thereby impairing antigen presentation to T cells and immunosurveillance. Thus, cytotoxic T cells may select for less immunogenic clones which have evolved such evasive maneuvers by eliminating more “visible” clones.

We have previously discussed the maturation block of dendritic cells in the context of bladder cancer and given their pivotal role in activating CD8 T cells, this too is a mechanism by which T cell cytotoxicity is restrained. Interestingly, some bladder cancer subtypes have an “immune desert” phenotype and evade immunosurveillance and CD8 mediated cytotoxicity by generating an environment which is hostile to them despite the presence of neo-antigens. A recent genomic analysis in bladder cancer found that activation of the PPARγ, FGFR3 and β-catenin pathways in a tumor correlated with poor immune cell infiltration despite a similar somatic mutational load to more heavily infiltrated tumors (8).

As activation of these pathways correlates to the molecular subtype of bladder cancer, this is confirmation of the previously discussed relationship between molecular subtype and immune infiltration. CD8 T cells lie at the end of a long story of activation and polarization of successive immune populations and, given this, any treatments such as checkpoint blockade which target them alone is likely to be limited in efficacy. In addition, their efficacy may be limited by adaptations of the tumor as described above. However, their incredible potential is demonstrated by the small cohort of patients who respond to checkpoint inhibition in urothelial cancer. Future therapeutic strategies would do well to combine multiple approaches along different axes, both innate and adaptive.

## Conclusion and Future Directions

**Figure 5:**
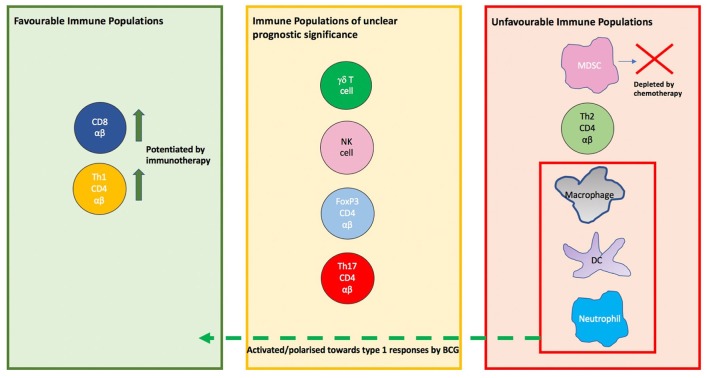
The prognostic significance of immune cell populations in the tumor and modulation by therapies. On the left are populations whose presence in tumor is associated with a favorable outcome such as CD8 cells and Th1 CD4 αβ T cells, with the activity of both potentiated by immunotherapy. On the right are populations whose presence in tumor is associated with tumor promotion. In the red box are populations which are shifted to a anti-tumor phenotype in the context of BCG therapy. In the center box are populations whose prognostic or predictive value in bladder cancer is unclear.

It is clear from the evidence considered that the immune system is highly active in the context of bladder cancer, however, some of these activities are greatly counter-productive and pro-tumorigenic. The tumor itself is a key immunological player, often profoundly shaping immune responses to favor itself. It is also clear that this is a multi-faceted system with contributions from the lymphoid and myeloid lineages (Figure 5). This explains the moderate success of therapies such as checkpoint blockage which target isolated axes within this system. Of note, innate cells of the myeloid lineage are often resident in the bladder and play an important role in shaping the immune response but are oft neglected in research and therapeutics. Future immunotherapeutic strategies have to focus on how the immunosuppressive tumor microenvironment may be curbed, in addition to enhancing the actions of innate immune subpopulations.

Whilst we have considered the extensive evidence for an overall suppression of immunosurveillance responses within the tumor, the understanding of tumor immunoediting *in vivo* is still a nascent study. However, with focus on antigen specific responses in future therapies such as CAR-T cells or peptide vaccines, understanding this process of clonal selection will become ever more important.

Additionally, the bladder is no longer thought to be a sterile organ and there is increasing interest in the urinary microbiome. Though there is some evidence from small studies of differences in the urinary microbiome between patients with bladder cancer and healthy individuals, the evidence is suggestive rather than definitive and no clear mechanisms have been identified (117). With the observation that antibiotic therapy appears to increase your risk of bladder cancer, there will no doubt be more interest in the impact of the urinary microbiome on pathogenesis and treatment for bladder cancer (118).

Perhaps, the most exciting avenues to explore involve combining the treatments which exist, such as BCG or checkpoint inhibition (which are currently being trialed in combination) with additional treatments to modulate the immune response for maximal effect. Thus, it may well be that every little helps in the anti-tumor response.

## Author Contributions

MJ planned and wrote the manuscript. DE was involved throughout in editing, review, and approval of the manuscript.

### Conflict of Interest

The authors declare that the research was conducted in the absence of any commercial or financial relationships that could be construed as a potential conflict of interest.
